# Comparison of public discussions of gene editing on social media between the United States and China

**DOI:** 10.1371/journal.pone.0267406

**Published:** 2022-05-02

**Authors:** Jiaojiao Ji, Matthew Robbins, Jieyu Ding Featherstone, Christopher Calabrese, George A. Barnett

**Affiliations:** 1 Department of Science and Technology Communication, University of Science and Technology of China, Hefei, China; 2 Department of Communication, University of California, Davis, California, United States of America; Fuzhou University, CHINA

## Abstract

The world’s first gene-edited babies event has stirred controversy on social media over the use of gene editing technology. Understanding public discussions about this controversy will provide important insights about opinions of science and facilitate informed policy decisions. This study compares public discussion topics about gene editing on Twitter and Weibo, as wel asthe evolution of these topics over four months. Latent Dirichlet allocation (LDA) was used to generate topics for 11,244 Weibo posts and 57,525 tweets from September 25, 2018, to January 25, 2019. Results showed a difference between the topics on Twitter versus Weibo: there were more nuanced discussions on Twitter, and the discussed topics between platforms focused on different areas. Temporal analysis showed that most discussions took place around gene-edited events. Based on our findings, suggestions were provided for policymakers and science communication practitioners to develop more effective communication strategies toward audiences in China and the U.S.

## Introduction

On October 7, the 2020 Nobel Prize in Chemistry was awarded to Emmanuelle Charpentier and Jennifer A. Doudna for discovering the advanced gene editing tool, CRISPR/Cas9, and its ability to transform lives [1]. Gene editing refers to a set of tools that biological researchers can use to modify specific genes (correct, introduce, or delete almost any DNA sequence) of living organisms [2]. CRISPR/Cas9, an acronym for Clustered Regularly Interspaced Short Palindromic Repeats and CRISPR-associated protein 9, is the most recent and widely used gene editing tool [2]. Compared to other gene editing methods, CRISPR/Cas9 can modify, delete, or correct precise regions of DNA in a faster, cheaper, more accurate, and more efficient way, which has revolutionized biomedical research and innovations [3].

However, gene editing has not always enjoyed acclaim. On November 25, 2018, the Chinese scientist He Jiankui posted a YouTube video announcing the birth of the world’s first gene-edited babies, which He and his team had helped to produce using CRISPR technology. His announcement came on the eve of the International Summit on Human Genome Editing in Hong Kong and was elicited, in part, due to public pressure from a post by Antonio Regalado, a senior editor for biomedicine of MIT Technology Review. The post revealed the problems of clinical trial registry in China and stirred public demand for Dr. He to explain if his human embryo experiment followed lawful procedures and ethical guidelines. The day after the video’s release, Chinese scientists condemned his unethical experiment, stating his action was “a huge blow to the global reputation and development of Chinese science” [4]. The event spread virally on U.S. social media, including Twitter, and started a wave of discussions about the ethics and government regulation of the technology [5]. While this event took place in China, few studies have looked at how Chinese social media sites reacted.

The discussion of scientific issues on social media has facilitated public understanding of scientific advancements [6], influenced public engagement in science policies [7], and created a diverse environment for scientific discussions [8]. For example, a survey in China found that the gene-edited babies event triggered public concerns over government regulation on clinical experiments and public interest toward the technology [9]. However, few studies have investigated the details of how the public reacted toward the ethics of bioresearch or discussed the technology on social media. For example, one study examining English tweets found that the gene-edited babies event drove discussions about gene editing technologies, however the authors only reported general themes rather than the specific details or topics of the discussions [5].

The content spread on social media may impact people’s attitudes and beliefs toward science and technology. For example, both Weibo and Twitter have facilitated discussions about global science and increased interest in scientific discoveries among their users [10]. Further, examining social media discussions may also help guide future public engagement strategies, which can help inform future policies related to the technology. Moreover, both the U.S. and China play critical roles in developing and applying gene editing technology, and are the two countries with the largest number of filed patents for CRISPR [11]. Therefore, comparing the discourse between two countries will help researchers understand how different publics discuss gene editing within their countries. This will help with the development of communication strategies and policies suitable for each country. Therefore, this paper seeks to analyze and understand the similarities and differences in public discussions surrounding gene editing on social media between the U.S. and China from a cross-cultural perspective. In doing so, this study contributes to cross-cultural research of public discussion regarding controversial science issues.

## Literature review

### Public engagement with science on social media: Twitter vs. weibo

Social media have become an integral part of the public engagement of science and technology, enabling participatory and reciprocal conversations and interactions between specialists and audiences [12, 13]. Specialists, including scientific communities, ethicists, and policymakers, increasingly use social media to open in-depth discussions about revolutionary technologies with the general public [14–16]. Social media have changed how the general public seeks information about societal issues and how they learn about the latest scientific advances [6]. Through social media, users express their engagement mainly through “content interactivity,” how users “control the information they receive” [17], and “human interactivity,” how users comment on content and share content with others [18]. The content on social media has been studied to examine different scientific issues, for instance, climate change [19] and vaccination [20, 21]. These studies have shown that social media have become important communication tools for scientific issues.

As a social media platform, Twitter serves as an important communication channel for the public engagement of science in the U.S. and worldwide. The majority of users are from the United States, with more than 47 million accounts [22]. For communicating science to broader lay audiences, Facebook and Twitter are of particular interest in the U.S. Although Facebook is the most popular social networking site [23], its platform features, such as restrictions on user activity to only accepted “friends,” make it difficult for researchers to connect with the public [24]. Unlike Facebook, tweets from unprotected accounts can be seen by everyone [25]. Further, Twitter discussions and real-time updates have the potential for the public engagement of science [24]. Consequently, numerous scientists from many disciplines use Twitter for public engagement [16].

Weibo, the equivalent of Twitter in China, is one of the largest social networking sites. Despite being the most popular microblogging service in China, there is limited research on how Weibo is employed for public engagement with science. For example, Yu et al. discovered that Weibo is an important local altmetrics platform on which global science is discussed [10]. Further, Rauchfleisch and Schäfer discussed how Weibo facilitated the information flow of sensitive topics, such as food safety and climate change [26].

Twitter and Weibo are similar in many aspects. Both are recognized as the top microblogging services with hundreds of millions of users worldwide [27]. The social networking elements are similar, and both enable users to post, repost, or forward messages [28]. Like Twitter, Weibo is also a directed social network, allowing users to follow other users and browse real-time trending topics. Both platforms heavily rely on advertising revenue [29].

Although Weibo has several similarities with Twitter, the social media platforms have different characteristics, which may influence public engagement with science. Twitter’s interface is less media-rich compared to Weibo. Twitter messages are limited to 280 characters and users can upload photos or short videos, while Weibo lets users insert animated emoticons, images, videos, music, and even polls. Furthermore, the types of trending topics on the two platforms are different. The trending topics on Weibo are often about entertainment and rarely about politics or science. While the trending topics on Twitter are more diverse; there are more discussions regarding science and politics [30, 31]. In addition, users’ demographics differ between the two platforms. U.S. Twitter users are about 40 years of age on average, highly educated, wealthier than the general public, and more likely to identify as a Democrat than a Republican [32]. However, 40% of Weibo users are 23 to 30 years old, and about 35% are aged 18 to 22 years [33].

### Scientific literacy and public discussion on social media

Social media, especially microblogging sites, have gained considerable academic attention for facilitating public discussion due to its open access and connected structure [34]. Social media have provided a space where privileged sectors and ordinary citizens, including small and marginalized groups, can create a public presence and discuss public issues. Public discussions cover a wide variety of topics, including family life, business, science and technology, and health [5, 35]. While people in different parts of the world may discuss the same issue, discussions may differ across countries [36].

Scientific literacy levels of engaged users may influence the public discussions of cutting-edge science issues on social media. Scientific literacy is considered one of the critical competencies for social inclusion, which will help any citizen access, read, and understand the world with a scientific dimension [37]. Various academic scholars argue that having a scientifically-informed population is essential for democratic processes in a technologically demanding society [38]. High literacy levels can lead to more reasoned and rational public discussions, which is vital for the informed decision-making process of legislators and industry [39]. These differed between the US and China [40]; for example, scientific literacy levels among Chinese citizens were relatively low compared to the U.S. and European countries in 1995 and 2005 [40]. Because of these differences, voices regarding gene-edited babies on Twitter could likely be distinct from those on Weibo.

Further, understanding perceptions of gene editing from a cross-country perspective allows researchers to examine how social media may shape the landscape of user opinions on science topics [6]. Since individuals from different cultural groups may receive and discuss different information online, they may have distinct resulting attitudes and opinions. Because social media is pervasive in our everyday lives, we can examine the primary topics that emerge from each platform and how they may shape or reinforce the platform users’ beliefs and opinions of gene editing. This understanding will help guide future message strategies for science communication.

### Discussions about gene editing

Human application of CRISPR have raised profound concerns, challenging scientific communities, bioethics groups, policymakers, and the public to think about the existing policy for governing human genome editing [41]. There are some concerns that gene editing science and innovation are moving ahead of public understanding and legislation [42]. It is urgent to call for public engagement and a broad public discussion about the ethical, legal, and wider societal implications of this technology.

Mainstream discussions regarding gene editing are usually about ethical and social concerns, including the safety of the technology, the alteration of the human genome, issues of eugenics, and regulation policies [43–45]. Though CRISPR has increased further specificity and reduced off-target effects, the risk of inaccurate editing is still a concern. He Jiankui has attempted to modify the CCR5 gene in embryos. Thus, there could be possibility that off-target changes in other genes were made in the twins’ genome [46]. Besides, once the mutations are introduced into the human population, the altered genes would be difficult to remove and may spread across communities or even countries [47]. The societal risks are as important as safety risks. Genetic enhancement of humans could result in social inequality and discrimination in society. Multiple polls indciate that there is greater support for therapy (disease treatment) than enhancement (e.g., improving memory and learning capacity) across different countries [48–51]. Many people oppose genetic enhancement in humans as it is intrinsically eugenic in nature; the inheritable genetic enhancement for “more desirable” and “better” kinds of humans [52].

Discussions on social media may influence the public’s knowledge and attitudes toward CRISPR/Cas9. Media have the power to construct public beliefs and attitudes about topics and events, especially when individuals have little prior knowledge of these subjects [53]. Since previous research has established the relationship between gene editing knowledge and science attitudes [54], examining the discussion of gene editing on social media is particularly useful. Several recent studies have provided some insights into online discussion about gene editing. For example, Calabrese et al. examined the discussions about CRISPR/Cas9 on Twitter in 2018, uncovering four main themes, including research and applications of CRISPR, the breaking news story of the use of gene editing on human embryos in China, biotechnology regulations in agriculture, and research relating to muscular dystrophy [5]. Further, the authors found that the “CRISPR babies” news story dominated discussions of gene technology, indicating that major events impact how users discuss socio-scientific issues in online media [5]. Walker and Malson undertook a textual analysis of Facebook comments on posts by news media outlets about agricultural and environmental gene editing and found four themes emerged, including gene editing as challenging a higher power, the conflation of gene editing with genetically modified organisms (GMOs), pro-science arguments, and comparisons to science fiction [55].

Because social media can shape people’s beliefs and attitudes toward new topics like gene editing, it is important to understand what users discuss on their respective platforms. Further, understanding the differences and similarities of how Chinese and American social media platforms discuss gene-edited babies, likely due to cultural differences, may promote mutual understanding and facilitate informed policy decisions. Moreover, discussions about scientific issues could dramatically change over time, especially during large events like the announcement of the first gene-edited babies. Examining the evolution of the topic distributions around the gene-edited babies event will help policymakers and science communication practitioners develop more effective communication strategies with the public. Thus, we propose the following research questions:

R1: What topics are discussed about CRISPR and gene editing on Weibo and Twitter?
R2: What are the differences of discussed topics between Weibo and Twitter?
R3: How do the discussed topics evolve over time?

## Methods

### Data collection

The terms “gene editing,” “genome editing,” “CRISPR,” and “CRISPR-Cas9” have been shown to be salient keywords on Twitter [5]. We conducted a preliminary search on Weibo using the corresponding Chinese keywords, and the majority of the obtained posts were closely related to gene editing technology. In addition to the above four terms, “genome engineering” is also widely used as “gene editing” and “genome editing” on Chinese social media. Therefore, to make the dataset comparable, we used “gene editing,” “genome editing,” “genome engineering,” “CRISPR,” and “CRISPR-Cas9” as searching keywords for Twitter, and “基因编辑,” “基因组编辑,” “基因组工程,” “CRISPR,” “CRISPR-Cas9” for Weibo. The search period was set two months before and after the Chinese gene-edited babies event to fully understand the public discussions, ranging from September 25, 2018, to January 25, 2019. Weibo and Twitter users have generated far more tweets on the gene-edited babies event during this period than ever before. We collected 11,244 posts from Weibo through Zhiwei, a well-known social media data provider, which collected post based on Weibo API, and 57,525 tweets from Twitter based on its Premium Search Tweets API. Weibo may have filtered some posts in compliance with the rules and laws of the Chinese government. Also, due to new Weibo functions that allow users to change their settings so that others only see posts within the most recent six months, some posts could not be collected.

### Data pre-processing

#### Pre-processing of weibo posts

Posts from Weibo were pre-processed before conducting topic modeling using Python, as shown in S1 Fig in S1 File. First, platform-specific processing measures were carried out, including removing all the URLs, user mentions, and duplicates. We did not track the trending hashtags specifically for the studied topic, but we kept the hashtags’ semantic content. Since there are no separators between words in Chinese text, Chinese lexical analysis is a prerequisite to Chinese information processing. Therefore, ICTCLAS (Institute of Computing Technology, Chinese Lexical Analysis System) was employed to tokenize the texts [56]. Also, the terms that should not be tokenized were predefined according to the context of gene editing: only nouns, verbs, and adjectives were selected from the cleaned corpus [57]. Then, stop words were removed from the corpus. Lastly, the corpus was normalized by using the created synonym dictionary so that matches would occur despite superficial differences.

#### Pre-processing of tweets

Tweets were similarly pre-processed before topic modeling using R, as shown in S2 Fig in S1 File. The standard procedures of language pre-processing were carried out, including tokenization of tweets to the word level, discarding punctuation and capitalization of words, pruning out a standard list of stop words and words occurring in only one tweet, and stemming. Platform-specific processing measures were also carried out in the form of removing URLs, user mentions, and duplicates. We removed the special character “#” but kept the hashtags’ semantic content.

### Topic modeling

Latent Dirichlet allocation (LDA) [58, 59], a widely used tool to extract topics from text data, was applied to Twitter tweets and Weibo posts. LDA is an unsupervised probabilistic model that generates mixtures of latent topics from a collection of documents, where each topic is characterized by a distribution over words. A distribution over topics is first sampled from a Dirichlet distribution, and a topic is chosen based on this distribution. LDA is a mathematical method for estimating both of these simultaneously: finding the mixture of words associated with each topic while also determining the mixture of topics that describes each document. Each document is modeled as a distribution over topics, with topics represented as distributions over words [60]. To find these topics, LDA uses word co-occurrence patterns in the corpus, such that the more often two words co-occur in a document, the more likely they are to be assigned to the same topic [61]. An important task for topic modeling is to determine the prior value for the number of topics (k); if k is too small, the topics will be overly broad, while if it is too large, the topics may be too overlapping or similar to each other.

To efficiently run topic modeling for larger datasets, we used LDA in this study. For tweets, the mean length is 119, and the median length is 131. For Weibo posts, the mean length is 116, and the median length is 142. The distributions for document lengths of tweets and Weibo posts are shown in S3 Fig in S1 File. Moreover, for topic quality, LDA can achieve a similar performance as other short text topic models like GSDMM and Biterm [62–64].

It is crucial to understand tweets and Weibo posts accurately. Hence, we applied two different topic models: one for tweets and one for Weibo posts. This has several methodological advatanges. First, due to the need to compare two different languages, English and Chinese, applying separate models removes issues relating to translating documents or the accuracy of the translations. Translation services are often not satisfactory for delivering accurate and context-specific translations, which may cause confusion. Because the U.S. and Chinese researchers in our team can fully understand and interpret the contents, utilizing two separate models is ideal. Second, applying a single cross-collection topic modeling, rather than two separate models, might allow some topics specific to one dataset to be overshadowed and ignored. For example, many of the topics on Weibo were specific to China and Chinese media, which may have been ignored with the addition of Twitter discussions. Third, we wanted to conduct a qualitative analysis of the differences between the U.S. and China and explain the differences from the communication perspective.

**Fig 1:**
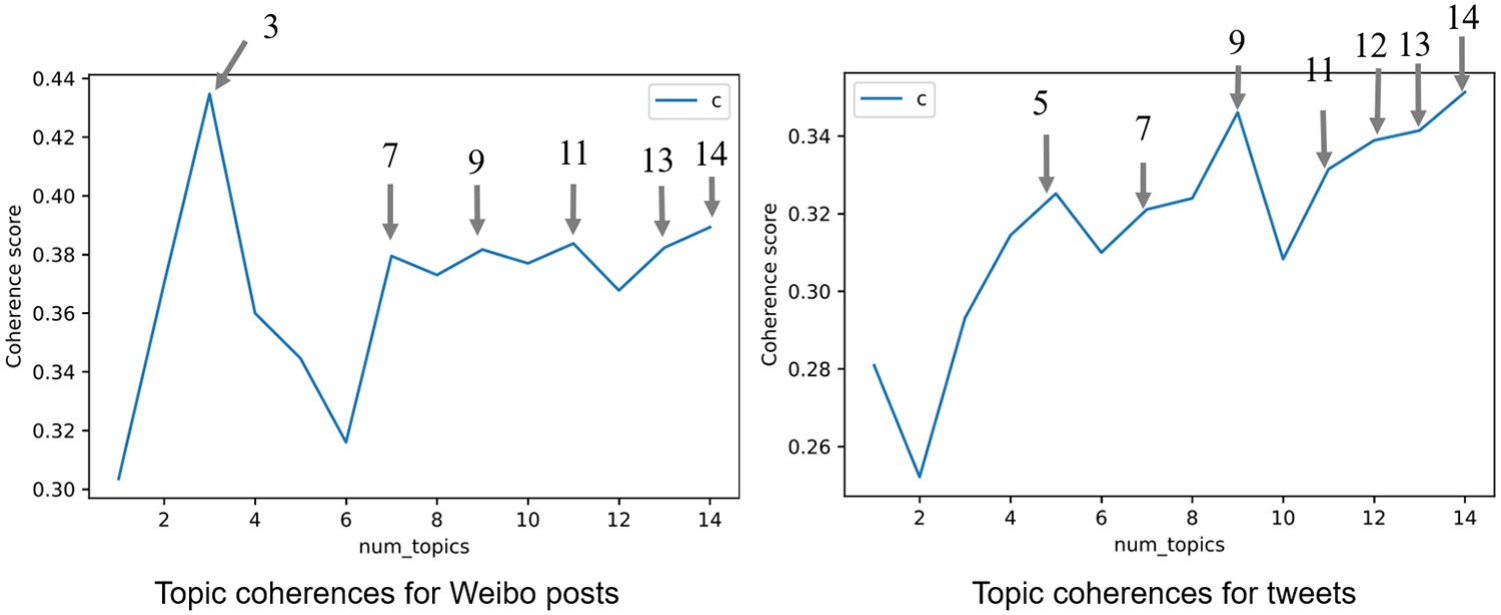
Coherence scores for weibo posts and tweets.

Following the steps recommended by [59, 65], we built and validated the models based on the following procedures. First, we selected the models based on the topic coherence score, which measures a topic’s semantic interpretability and association with well-defined semantic concepts [66]. As a high score denotes meaningful and interpretable topics, we trained multiple LDA models and selected models with relatively high coherence values at peaks (Fig 1). For Weibo posts, we chose the models when k was 3, 7, 9, 11, 13, and 14; For tweets, we selected the models when k was 5, 7, 9, 11, 12, 13, and 14. Second, researchers interpreted the topics based on the most frequent keywords and a sample of documents with the highest probability. During the process of interpreting, we also used intertopic distance mapping to assist with the determination of topic selection. The distance map was drawn and visualized using the Python package pyLDAvis [67], where the distances between topics are first calculated using Jensen-Shannon divergence, and then the intertopic distances are projected onto two dimensions [68]. If they were overlapping, the semantic contents of the topics would be similar. Based on interpretation and intertopic distance map, we chose models that were well interpretable and not overlapping, as shown in S4 Fig in S1 File. Finally, we re-evaluated the validity of interpretation by checking a sample of automatically assigned documents. S1 and S2 Tables in S1 File list the reasons why we did not accept other models. Therefore, we set the number of topics as 3 for Weibo posts and 9 for Twitter. The example tweets for 9 topics and example Weibo posts for 3 topics are listed in S3 and S4 Tables in S1 File.

## Results

**Fig 2:**
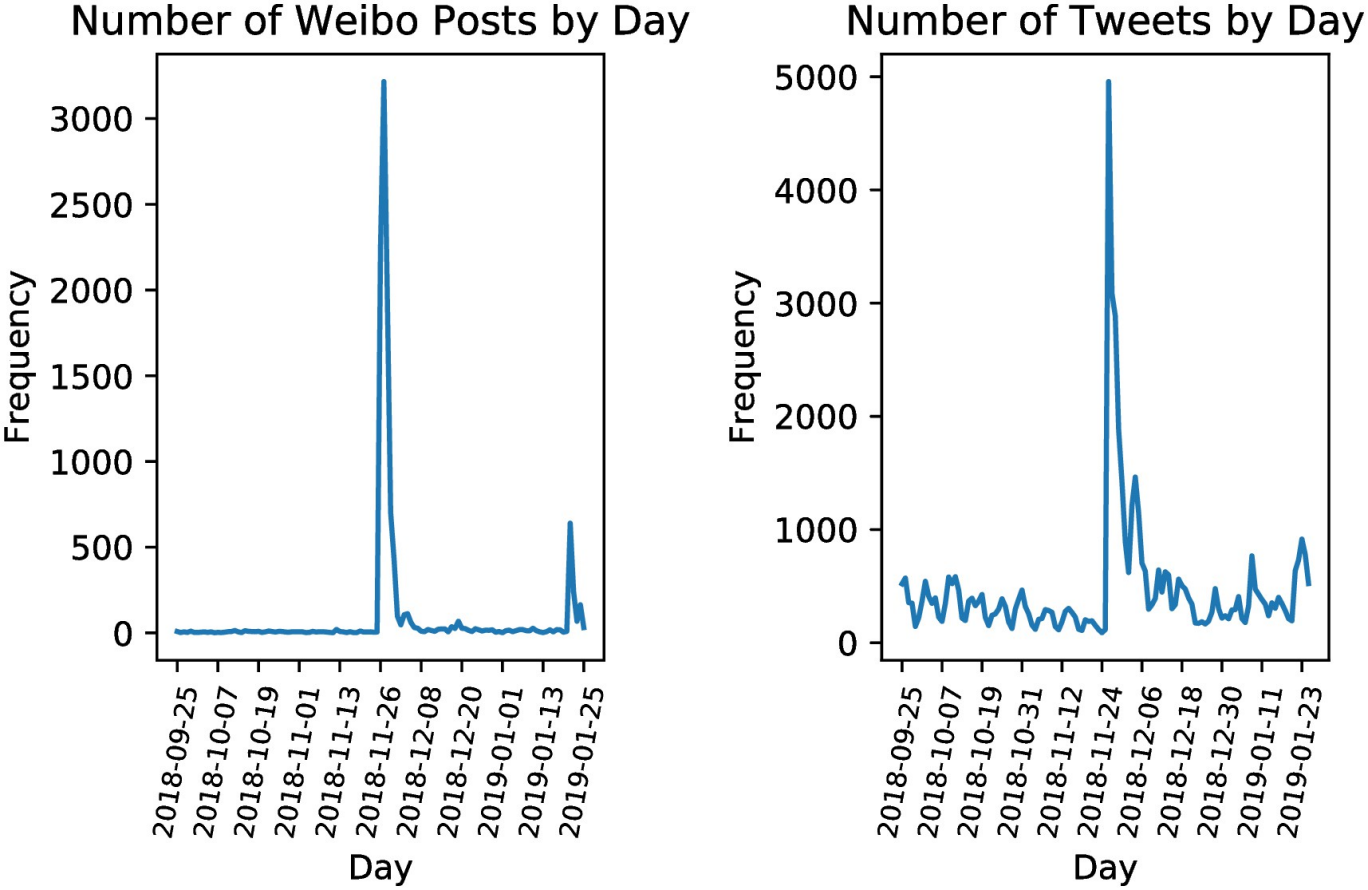
Distribution of posts about gene editing from September 25, 2018 to January 25, 2019.

Fig 2 depicts the number of Weibo posts and tweets posted each day from September 25, 2018, to January 25, 2019. He Jiankui announced the birth of twin girls with edited genomes on November 25, 2018. For both Weibo and Twitter, the majority of posts were posted between November 26 and December 5, 2018. Compared with Weibo, there are more small peaks during the four months, indicating there was more attention to gene editing on Twitter.

### Topics on weibo

As is shown in Table 1, there were three topics about gene editing during this period on Weibo. Topic 1 talks about the Chinese government’s investigation into Dr. He Jiankui, the Associate Professor from Southern University of Science and Technology (SUST) (Guangdong is the province that includes the city of Shenzhen, where SUST and the Hemei Hospital that allegedly gave ethics permission for the experiment is located). Chinese authorities from the Guangdong province placed Dr. He under investigation and claimed that his work defied government bans and was “in the pursuit of personal fame and profit.” However, they also found that Dr. He “deliberately evaded supervision, raised funds and organized researchers on his own to carry out the human embryo gene-editing intended for reproduction, which is explicitly banned by relevant regulations [69].” This topic accounted for 21.8% of the tokens.

**Table 1:** Topics for Weibo posts based on LDA.

Topic#	Topic Words—ranked by their probabilities in topic	Topic label	Percentage of tokens
**1**	find out, He Jiankui, investigation team, activities, Guangdong province, science and technology, Southern University of Science and Technology, Guangdong, investigation, ban, implementation, purpose, fame and profit, organization, human, supervision, embryonic gene, associate professor, funding, evade, edit, self-funded, response, reproduction	The Chinese investigation into He Jiankui	21.8%
**2**	gene editing, clone, gene, technology, monkey, CRISPR, science, somatic cell, world, human, research, biology, model, gene editing technology, utilization, scientist, rhythm, ethics, United States, success, disorder, therapy, clinical, birth, disease	Scientific aspects of gene-editing technology	33.9%
**3**	He Jiankui, HIV immunity, human, gene editing, birth, Summit, gene, scientist, world, first case, Shenzhen, international, ethics, genome editing, AIDS, project, birth, hospital, controversy, experiment, speech, research, experiment, response, Nana	Gene-edited baby controversy discussed around the International Summit on Human Genome Editing.	44.3%

Topic 2 concerns scientific aspects of CRISPR technology, especially the achievements based on the use of the technology. One of the most popular achievements discussed on Weibo was on January 24, 2019, where Chinese scientists cloned five monkeys from a gene-edited macaque by somatic cell nucleus transfer (SCNT). Clones produced this way may be useful for the development of non-human primate models of human diseases (circadian rhythm disorders). Another news story is from XinhuaNet where a U.S. genetics company announced the launch of a clinical trial using CRISPR to treat hereditary eye disease. Topic 2 involved a third (33.9%) of the tokens.

Topic 3 concerns the International Summit on Human Genome Editing controversy at the University of Hong Kong. It involved the most tokens, 44.3%. Dr. He was invited to speak at the Summit and described how his research team had conducted experiments on cultured human embryos to disable a gene known as the CCR5 gene in an attempt to confer genetic resistance to human immunodeficiency virus (HIV). According to the percentage of the tokens, most of the posts on Weibo are about the Summit in Hong Kong.

### Topics on twitter

Nine topics about gene editing were discussed on Twitter, as shown in Table 2. It also lists the percentage of tokens for each topic. Topic 1 depicts the commercial aspects of CRISPR technology, such as the market value of CRISPR technology, the market share of the biotech companies (CRISPR Therapeutics AG), and the CRISPR/Cas9 intellectual property battle. Topic 2 is about biohacking CRISPR technology, by one famous biohacker, Josiah Zayner, who runs a company called the Odin out of his garage in Oakland, California, selling biohacking supplies, including DIY kit. He injected himself with DNA from CRISPR at a biotech conference. Topic 3 emphasizes that CRISPR is a powerful research tool, especially its targeting specifics. These tweets are usually about scientific research from academic journals, such as *Nature Biotechnology*, *bioRxiv*.*org*, and *Nature Cell Biology*.

**Table 2:** Topics for tweets based LDA.

Topic #	Topic Words—ranked by their probabilities in topic	Topic label	Percentage of tokens
**1**	therapeutic, 2019, fellow, therapy, biotech, crsp, exclusive, patent, deal, biggest, behind, company, biotechnology, million, market	Application of CRISPR technology to Therapy Market	6.2%
**2**	track, biohack, kit, fast, flu, toward, time, learn, meet, intelligence, artificial, blockchain, office, agriculture, little	CRISPR Science biohacking	3.5%
**3**	cas9, genome, cell, develop, tool, control, base, target, precise, guide, system, lab, screen, 2018, revolutionary	CRISPR technology application in research	15.4%
**4**	engineering, one, like, science, story, know, explain, change, today, great, want, repurpose, video, food, decade	Science communication of CRISPR technology	11.6%
**5**	year, face, death, penalty, modify, geneticist, fire, controversy, people, take, good, point, go, possible, edit	Chinese scientist who claims to have created the world’s first genetically edited babies could face death penalty	8.7%
**6**	disease, antibiotics, better, study, hope, fail, resist, mutation, born, glimmer, inherit, immune, govern, locus, get	Advantages of CRISPR	7.4%
**7**	Chinese, create, scientist, claim, universe, tomato, report, spicy, made, say, review, investing, clone, chili, pest	Development of genetically modified spicy tomatoes	7.3%
**8**	switch, warn, scientific, cancer, medicine, potential, HIV, application, trial, patient, simple, medical, clinic, risk, public	Clinical application of CRISPR technology	8.1%
**9**	edit, gene, baby, use, scientist, genetic, first, human, China, research, technology, world, work, make, ethic	Ethics of the human application of CRISPR, including world’s first gene-edited babies	31.8%

Topic 4 involves science communication about CRISPR technology. Many tweets share a link to a popular video on YouTube named *Genetic Engineering Will Change Everything Forever*, which introduces CRISPR. Also, a large number of tweets linked to an article from *Vox* titled “A simple guide to CRISPR, one of the biggest science stories of the decade.” Topic 5 discusses the consequences of conducting controversial experiments. For example, one tweet stated, “CRISPR-baby scientist fired by the university.” Geneticist Robin Lovell-Badge of the Francis Crick Institute in London was worried that Dr. He could face the death penalty [70]. Topic 6 focuses on the advantages of CRISPR technology, and mainly relates to two news articles. One article is titled, “Antibiotics Are Failing Us. Crispr Is Our Glimmer of Hope #AntibioticResistance”, and the other is titled, “CRISPR Repurposed to Develop Better Antibiotics.” According to Rettedal (2019), “antibiotics are still massively overprescribed, a new study shows. With no new drugs in sight, some scientists are turning to CRISPR for a reboot” [71].

Topic 7 strengthens the development of genetically modified spicy tomatoes. Despite this controversy surrounding He Jiankui, scientists were still interested in CRISPR and might soon create spicy tomatoes by switching on their chili genes, according to MIT Technology Review. Topic 8 describes the clinical application of CRISPR, especially its use for the treatment of brain cancer and for medicine. Topic 9 has the largest proportion of the tokens and focuses on the ethics of the human application of CRISPR, including the world’s first gene-edited babies. This is most likely due to the period during which the posts were collected.

### Topic distributions over time

**Fig 3:**
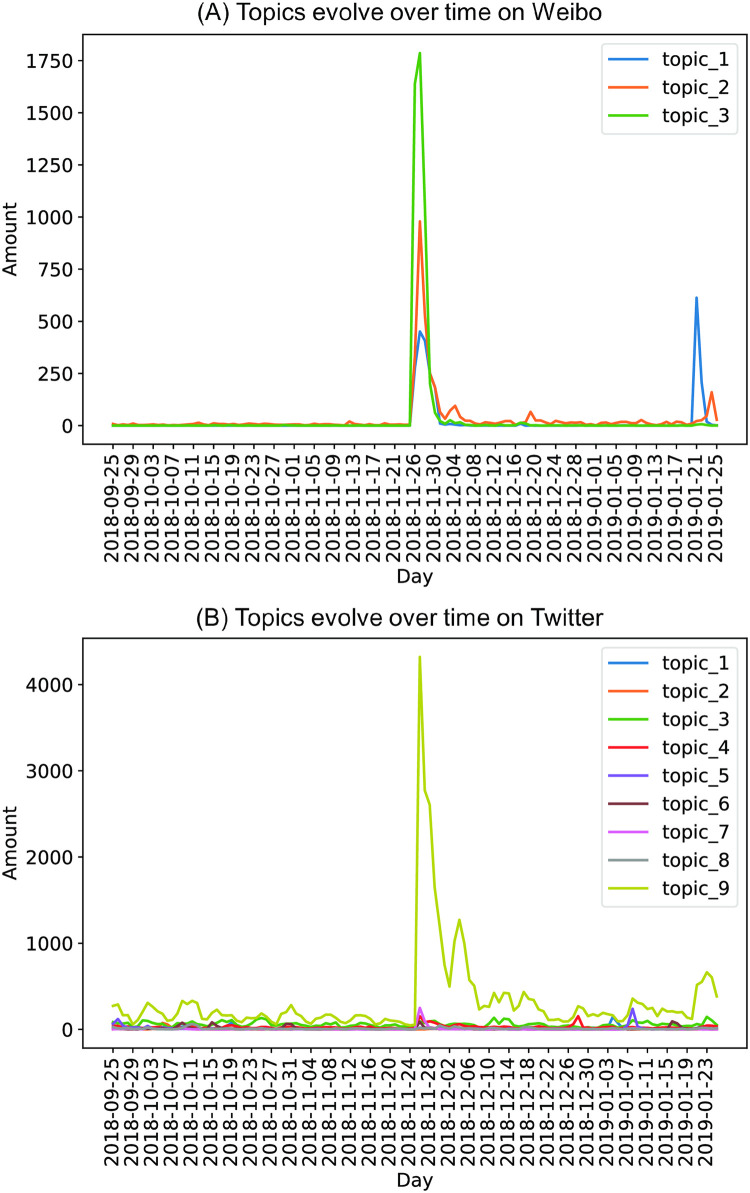
Temporal distribution of topics on Weibo (A) and Twitter (B).

Fig 3 depicts the evolution of the topic distributions over time on Weibo and Twitter. Dr. He announced the birth of twin girls with edited genomes on November 25, 2018. The topics peaked on November 27, 2018 (Beijing time) on Weibo and peaked on November 26, 2018 (New York time) on Twitter. The discussion of these topics then decreased rapidly, indicating the discussion of CRISPR technology was primarily driven by the designer babies event. On Weibo, most of the posts were about the gene-edited baby controversy discussed at the Summit, and the least was about the investigation into Dr. He’s research on November 26, 27, and 28, 2018. Then, gene editing quickly faded from public attention, and only a few posts mentioned the scientific aspects of CRISPR technology. However, the public discussed the investigation into Dr. He’s research again in January 2019. On Twitter, people seldom discussed gene editing before November 25; however, all the topics peaked on November 26. Generally, more attention was paid to gene editing after the event. The most discussed topic was the ethics of designer babies, which peaked on November 26, 2018. Another highly discussed topic was communicating the science of CRISPR technology. The event of gene-edited babies by Dr. He may have stimulated public interest in gene editing, and many people may be starting to learn about this technology.

## Discussion

Twitter and Weibo are vital platforms for public engagement with science. The discussions on these platforms may potentially influence public perceptions of gene editing and the process of policy decision-making. Consequently, it is of great importance to understand the contents of gene editing posts on social media. This study compares gene editing discussions on Twitter and Weibo by exploring the key topics that emerged over time through topic modeling analyses.These findings will help researchers understand perceptions of gene editing from a cross-cultural perspective and how social media may shape the landscape of user opinions on science topics.

The topics on Twitter and Weibo differed; a higher number of topics were discussed on Twitter, and the discussed aspects focused on different areas. Topics on Weibo were mainly centered around the International Summit and investigation into Dr. He’s activities. At the same time, people also posted about the techniques used and applications in stem cell research. For topics on Twitter, most of the discourse surrounding around the ethics of CRISPR’s human applications, especially the ethics of the world’s first gene-edited babies. Twitter users also mentioned scientific aspects, including CRISPR in research, biohacking, and CRISPR’s advantages. Different from topics on Weibo, there was evidence of science communication of gene editing on Twitter. Many tweets explained or introduced gene editing by including links to other websites. Also, Twitter discussed the application of CRISPR to therapy, clinical use, and scientific research.

Several reasons may justify the topic differences between Weibo and Twitter. The first involves the differing levels of scientific literacy. Because the U.S. has lead in the development and applications of gene editing technologies, regulations regarding human gene editing are more well-established, and people began discussions on the issue earlier [48]. Also, as aforementioned in the literature review, users’ demographics differ between two social media platforms. For example, U.S. Twitter users are about 40 years of age, highly educated, wealthier than the general public, and more likely to identify as a Democrat than a Republican [32]. Consequently, users on Twitter could be more familiar with gene editing and discuss more aspects, including clinical application, advantages of the technology, and science communication. This may explain why the discussed topics on Weibo were less wide-ranging than on Twitter. The second reason for the difference between Weibo and Twitter may be related to cultural differences. People from Western cultures like the U.S. are usually highly individualistic: they may emphasize uniqueness and hold independent views, while people from Eastern Asian cultures, like China, often may follow collectivism: they tend to value interdependence and hold interdependent views [72–74]. Thus, public discussion topics regarding the new technology would be more diverse on Twitter than on Weibo.

The evolution of topic distributions between the two platforms was slightly different. The temporal analysis shows that almost all the topics peaked on Twitter and Weibo from November 26 to 30. Over five days, the world’s first gene-edited babies were announced, and the International Summit on Human Genome Editing took place at the University of Hong Kong. The discussion was strongly influenced by the gene-edited babies controversy on both platforms. Before and after the gene-edited babies event, Weibo users seldom discussed gene editing, and they focused on the event for a short period. On Twitter, there was some discussion before the event. After the event, while the issue of gene editing babies largely disappeared from the public space, discussions about gene editing still lingered. One possible explanation for different evolution patterns is that the sources of the posts were different. Weibo is more of a platform for marketing, advertising, and entertainment than public discussion [75]. Therefore, Weibo users may prefer entertainment-related content to news and scientific topics. Also, the majority of the discussions were from verified accounts, who usually pay attention to major current events and shift their attention quickly when an important new event happens. Hence, Weibo users’ interest in gene editing did not last as long as Twitter users.

Further, the sources of posts were different between the two platforms. On Weibo, the majority of the accounts talking about gene-edited babies were from verified accounts (see S5A Fig in S1 File; however, the primary discussion on Twitter came from unverified users (see S5D Fig in S1 File. For verified users on both Weibo and Twitter, their accounts are authentic and notable [76, 77], and they usually enjoy higher reputations on social media. Besides, the percentages of unverified users on Twitter and Weibo are much higher than verified users. Compared to Twitter unverified users (see S5E Fig in S1 File, Weibo unverified users were less engaged in public discussion (see S5B Fig in S1 File. Science communication practitioners should encourage lay audiences on Weibo to get more involved in public discussion of gene editing. The distribution of topics differed between unverified and verified users on Weibo (see S5C Fig in S1 File; however, the distribution was the same on Twitter (see S5F Fig in S1 File. Weibo’s unverified users mainly centered on scientific aspects, indicating they were likely unaware of ethical problems and social impacts. Also,verified users on Twitter may be much more influential than those on Weibo. Therefore, it would be vital to select those opinion leaders that may have an impact on the behaviors of the lay public.

Weibo verified users should discuss other aspects of gene editing instead of concentrating on the gene-edited babies’ event. Meanwhile, Twitter users should pay more attention to other topics rather than purely focusing on the ethics of gene editing, which accounted for a relatively large proportion of tweets over time. The application of CRISPR and its advantages were not mentioned as much comparatively. For both platforms, the pros and cons of gene editing technologies should be fully addressed. Overemphasizing ethics rather than encouraging discussions surrounding these issues may hinder the process of innovation.

The rise of the topic about science communication on Twitter and science aspects on both Weibo and Twitter may indicate that the gene-edited babies claim may have driven the process of science communication. The gene-edited babies event was the first introduction to human genome editing for many social media users. Although CRISPR was discovered in 1993 and developed for more than twenty years, the public had never before discussed the technical, societal, and ethical issues in depth on such a large scale. Particularly in China, gene editing may not have been widely discussed [51]. Although only three topics were found on Weibo, discussions greatly changed in China. This event has introduced the technology to the public, and many people also started learning about it. Accordingly, science communicators could have seized the opportunity to communicate science to the public when they were curious about gene editing. Having more informed users would facilitate rational public discussions, which will guide decisions regarding the development of policies [78].

Topic differences between Twitter and Weibo may represent how Chinese and U.S. cultural values play a role in emphasizing specific aspects of gene editing on social media. By acknowledging how cultural values may play a role in how social media discussions are formed, science communication scholars can gain a deeper understanding of how to further engage with their public. Future research should examine the portrayal of gene editing among other countries to provide a more holistic view on why certain aspects of a scientific topic may be emphasized and how they can be addressed. It is increasingly important to engage with our different audiences through tailored messaging to ultimately further develop scientific innovation and policy decisions.

We removed the user mentions in the preprocessing of datasets because this study focused primarly on the users’ message contents about gene editing. However, the user mentions may contain meanings, and may help us understand the association between the human hub and related topics. While beyond the scope of our study, future work should focus on analyzing mention networks and their potential influence on social media with regard to topics like gene editing technologies.

## Conclusion

This study compared the topics of public discussions regarding gene editing, as well as the evolution of topics over four months on Twitter and Weibo based on LDA topic modeling. Our findings indicated that the topics on Twitter are more diverse than on Weibo. Weibo focused more on the investigation of Dr. He and the controversy over the event but less on the application of the technology itself. In contrast, Twitter included all topics discussed on Weibo and expanded the application of gene editing to different areas, such as therapy, agriculture, and biohacking. Moreover, temporal analysis revealed that most discussions took place from November 26 to November 30, and the public showed great interest in gene editing during this short period. This indicates that science communication practitioners should take advantage of major events to timely communicate science effectively to the public. Further, the differences between individualistic and collectivistic cultures may explain the difference in Twitter and Weibo discussions. The information presented on these different platforms will better inform science communication researchers to develop tailored message strategies that address the specific issues and concerns surrounding gene editing. Finally, the government should improve their communication with the public about scientific issues on social media, which will benefit the process of policy decision making.

## Supporting information

S1 File (DOCX)
