# Supplementary material for: Comparison of public discussions of gene editing on social media between the United States and China
Source: PLoS One. 2022 May 2;17(5):e0267406. doi: 10.1371/journal.pone.0267406 (PMC9060334; doi:10.1371/journal.pone.0267406)
Supplement: S1 File — (DOCX) [file pone.0267406.s001.docx]

**Comparison of Public Discussions of Gene Editing on Social Media**

**between the United States and China**

Table of contents

[**S1 Table 1. Reasons for non-acceptance of topics on Weibo.** 2](#_Toc101511918)

[**S1 Table 2. Reasons for non-acceptance of topics on Twitter.** 3](#_Toc101511919)

[**S1 Table 3. Example tweets for topics on Twitter.** 4](#_Toc101511920)

[**S1 Table 4. Example Weibo posts for topics on Weibo.** 6](#_Toc101511921)

[**S1 Fig 1. Preprocessing steps for Weibo posts.** 8](#_Toc101511922)

[**S1 Fig 2. Preprocessing steps for tweets.** 8](#_Toc101511923)

[**S1 Fig 3. The distributions for document lengths of tweets and Weibo posts.** 9](#_Toc101511924)

[**S1 Fig 4. Intertopic distance mapping for Weibo posts (left) and tweets (right).** 10](#_Toc101511925)

[**S1 Fig 5. Topic difference between verified accounts and unverified accounts.** 10](#_Toc101511926)

# **S1 Table 1.** **Reasons for non-acceptance of topics on Weibo.**

| Number of topics (k) | Reasons for non-acceptance |
| --- | --- |
| 7 | According to the intertopic distance map, topics 1 and 6, topics 5 and 7 were almost overlapping with each other. Moreover, their semantic contents were identical. |
| 9 | The semantic content of topic 4 was hard to interpret. Topic 5 and topic 8 are overlapping on the intertopic distance map, and topic 3 and topic 9 are semantically identical. |
| 11 | Topic 6 was hard to interpret. Besides, according to the intertopic distance maps, topic 2 and topic 8 overlapped, and topic 3 and topic 9 overlapped. |
| 13 | Topic 1 and topic 2 were semantically very similar. Also, there was an overlap between topic3, topic8, and topic9 on the intertopic distance map. Topic 4, topic 6, and topic 12 were hard to interpret. |
| 14 | Topic 1 and topic 11 were semantically very similar. Topic 2, topic 3, and topic 9 had identical meanings. And topic 5 and topic12 overlapped on the intertopic distance map. |

# **S1 Table 2.** **Reasons for non-acceptance of topics on Twitter.**

| Number of topics (k) | Reasons for non-acceptance |
| --- | --- |
| 5 | Topics 2 & 4 and topics 3 & 5 overlapped on the intertopic distance map. |
| 7 | There were overlaps among topic 2, topic 7, and topic 5. Besides, the semantic contents of these three topics were hard to interpret. |
| 11 | There were many overlaps among topics. Only topics 4, 6, & 8 were independent on the map. Topics 1, 5, 10, & 7 were overlapping. Topics 2 and 11 overlapped. Also, topics 3 & 9 overlapped. |
| 12 | 10 out of 12 topics in this model overlapped. Only topics 6 & 9 were independent. |
| 13 | Only three topics were independent, and the rest were overlapping. |
| 14 | 9 out of 14 topics were overlapping and semantically uninterpretable. |

# **S1 Table 3.** **Example tweets for topics on Twitter.**

| Topic ID | Topic | Example tweets |
| --- | --- | --- |
| 1 | therapeutic, 2019, fellow, therapy, biotech, crsp, exclusive, patent, deal, biggest, behind, company, biotechnology, million, market | Up to $818 million deal between J& J and Locus Biosciences points to a new path for CRISPR therapies \| The up to $818 million deal between Locus Biosciences and Janssen Pharmaceuticals (a division of Johnson &amp; Johnson) that was... \| https://t.co/KAZK0aH4gc https://t.co/rraBk0YMJf |
|  |  | Biotech firm to use CRISPR patents in rodent models. Biotech firm genOway acquired the exclusive global rights to Merck Group’s CRISPR patents. https://t.co/lAVyFSk785 #Biotech #CRIPR #genOway #patent #lifesciences |
| 2 | track, biohack, kit, fast, flu, toward, time, learn, meet, intelligence, artificial, blockchain, office, agriculture, little | learn #CRISPR protocols and how to set up CRISPR experiments with the guided #DIY kit from https://t.co/WhL4XF38sj and use the coupon code tryptophan for 10% discount. #biohacking #biohacker #Gentechnik #science #plantscience #agriculture |
|  |  | the kits from odin lead to a deeper understanding of theoretic taskes like the effects of a base substitution performed by #crispr #cas9 so if you want to enter the #biohacker community you should get a kit from odin #biotech #crispr |
| 3 | cas9, genome, cell, develop, tool, control, base, target, precise, guide, system, lab, screen, 2018, revolutionary | Genome-wide target specificities of #CRISPR RNA-guided programmable deaminases. #Base_Editor  Kim D, Lim K, Kim ST, Yoon SH, Kim K, Ryu SM, Kim JS.  Nat Biotechnol. 2017 May;35(5):475-480. Online 2017-04-10  https://t.co/Zy5YkYgdvU |
|  |  | New plasmids from the lab of @jonathan_ploski .  The Development of an #AAV-Based #CRISPR SaCas9 #GenomeEditing System That Can Be Delivered to #Neurons in vivo and Regulated via Doxycycline and Cre-Recombinase https://t.co/UeIcuGZ6JJ https://t.co/7f3Vpa05no |
| 4 | engineering, one, like, science, story, know, explain, change, today, great, want, repurpose, video, food, decade | A simple guide to CRISPR, one of the biggest science stories of the decade https://t.co/vweV2XLcyE https://t.co/YpSShbKSNO |
|  |  | #SciComm student Ellasin Chandrasma made this video to explain CRISPR as part of her studies here at Sheffield. We thought we would share as there has been much discussion over the last few days regarding gene editing in humans.  https://t.co/Op8DXEGWsz |
| 5 | year, face, death, penalty, modify, geneticist, fire, controversy, people, take, good, point, go, possible, edit | Wait, he’s missing? Thought he was under guard?: Chinese Scientist Who Created CRISPR Babies Could Face the Death Penalty, Fellow Geneticist Warns&lt;em&gt;&lt;/em&gt; https://t.co/StH8XGMXSi via @gizmodo |
|  |  | He Jiankui Fired in Wake of CRISPR Babies Investigation https://t.co/zE8p6ljvUH via @GENbio |
| 6 | disease, antibiotics, better, study, hope, fail, resist, mutation, born, glimmer, inherit, immune, govern, locus, get | Antibiotics Are Failing Us. Crispr Is Our Glimmer of Hope #AntibioticResistance https://t.co/gL5CSXTTNU |
|  |  | #CRISPR cures inherited #disorder in mice, paving way for #genetictherapy before birth https://t.co/AlcQ7Arbh0 via @statnews  CRISPR cures inherited disorder in mice, paving way for genetic therapy before birth  https://t.co/5hXMthoy40 |
| 7 | Chinese, create, scientist, claim, universe, tomato, report, spicy, made, say, review, investing, clone, chili, pest | Tomatoes and chiles have a common ancestor, and now they could be reunited .. to get spicy tomatoes!  CRISPR might soon create spicy tomatoes by switching on their chili genes https://t.co/c4eDaQaFW1 via @techreview |
|  |  | CRISPR might soon create spicy tomatoes by switching on their chili genes - MIT Technology Review https://t.co/yWb0ojANRK, see more https://t.co/88zgHuEwGc |
| 8 | switch, warn, scientific, cancer, medicine, potential, HIV, application, trial, patient, simple, medical, clinic, risk, public | Brain Cancer’s ‘Immortality Switch’ Turned Off with CRISPR https://t.co/QiOPw74tTi |
|  |  | Engineering CRISPR guide RNA riboswitches for in vivo applications. https://t.co/6peRot3qEZ |
| 9 | edit, gene, baby, use, scientist, genetic, first, human, China, research, technology, world, work, make, ethic | #Tech “Work on world’s first CRISPR gene-edited babies declared illegal by China https://t.co/8KjdmT4Kqb by ritacyliao https://t.co/FqLnH6WPw5” #Technology  Work on world’s first CRISPR gene-edited babies declared illegal by China https://t.co/8KjdmT4Kqb by ritacyliao pic.twitt |
|  |  | First gene-edited babies claimed in China. A Chinese researcher claims that he helped make the world’s first genetically edited babies -- twin girls whose DNA he said he altered with CRISPR. via /r/worldnews https://t.co/y38PUTaLzl |

# **S1 Table 4.** **Example Weibo posts for topics on Weibo.**

| Topic ID | Topic | Example Weibo posts |
| --- | --- | --- |
| 1 | 查明(find out), 贺建奎(He Jiankui), 调查组(investigation team), 活动(activities), 广东省(Guangdong province), 科技(science and technology), 南方科技大学(Southern University of Science and Technology), 广东(Guangdong), 调查(investigation), 明令禁止(ban), 实施(implementation), 目的(purpose), 名利(fame and profit), 组织(organization),人类(human), 监管(supervision), 胚胎基因(embryonic gene), 副教授(associate professor), 资金(funding), 逃避(evade), 编辑(edit), 自筹(self-funded), 回应(response), 生殖(reproduction) | 【南科大发声：解除与贺建奎的劳动合同关系】南科大官网1月21日发布声明称，校方研究决定：解除与贺建奎的劳动合同关系，终止其在校内一切教学科研活动。稍早时候，广东省“基因编辑婴儿事件”调查组公布调查结果：贺建奎伪造伦理审查书，指使人员违规操作，将依法依规严处，涉嫌犯罪的将移交警方。 |
|  |  | 【基因编辑婴儿事件后续：贺建奎“青年科技奖”参评资格被取消】中国科协党组书记、常务副主席怀进鹏表示，中国科协将进一步加大面向科技界的科研伦理道德的教育力度，以“零容忍”的态度处置严重违背科研道德和伦理的不端行为，取消贺建奎第十五届“中国青年科技奖”参评资格。http://t.cn/EL8KWuU ​ |
|  |  | //@伊春中院:【46名律师联名发声：建议公安机关立即对贺建奎采取强制措施】11月26日，46名律师联合发布声明谴责贺建奎。声明称，基因编辑婴儿行为不仅违反生物医学伦理，更违反法律价值所保护的“公平正义”，建议公安机关立即对贺建奎立案侦查并采取强制措施，全面调查此基因编辑婴儿事件的各方责任人 |
| 2 | 基因编辑 (gene editing), 克隆(clone),基因(gene), 技术(technology),猴(monkey), CRISPR (CRISPR),科学(science),体细胞(somatic cell),世界(world),人类(human),研究(research),生物(biology),模型 (model),基因编辑技术(gene editing technology), 利用(utilization),科学家(scientist),节律(rhythm),伦理(ethics),美国(United States), 成功(success),紊乱 (disorder), 治疗(therapy), 临床 (clinical), 诞生(birth), 疾病 (disease) | 【世界首例生物节律紊乱体细胞克隆猴模型诞生】#科技动向#中国科学院神经科学研究所的生物节律与衰老疾病研究组和非人灵长类研究平台经过两年努力，利用CRISPR/Cas9技术，成功构建了世界首例核心节律基因BMAL1敲除食蟹猴模型。该项研究成果，填补了生物节律紊乱研究高等动物模型的空白，突破了利用活体 ​ |
|  |  | 【中国科学家创建世界首例生物节律紊乱体细胞#克隆猴#模型】继2017率先攻克非人灵长类动物体细胞核克隆这一世界性难题、成功诞生世界上首个体细胞克隆猴“中中”和第二个克隆猴“华华”之后，中国科学家首次利用基因编辑方法，并通过体细胞克隆技术，获得5只生物节律核心基因BMAL1敲除的克隆猴，在国际 ​ |
|  |  | 【中国科学家成功克隆出杂交稻种子】中国农科院中国水稻研究所水稻生物学国家重点实验室王克剑团队利用基因编辑技术，建立了水稻无融合生殖体系，成功克隆出杂交稻种子，令杂交稻性状可以稳定遗传到下一代。“这个工作证明了杂交稻进行无融合生殖的可行性，是无融合生殖研究领域的重大突破，具有重大的 ​ |
|  |  | 【基因编辑人体临床试验将在美国启动，非生殖细胞不涉及遗传】美国一家基因编辑公司近日宣布，将启动一项利用CRISPR基因编辑技术治疗某种遗传性眼疾的临床试验，相关申请已被美国监管部门接受。在这一临床试验中，基因编辑的对象是先天性黑朦病患者眼睛里的感光细胞，这是一种体细胞，而非生殖细胞。体 ​ |
| 3 | 贺建奎 (He Jiankui), 免疫艾滋病(HIV immunity), 人类(human),基因编辑(gene editing),诞生(birth), 峰会(Summit), 基因(gene), 科学家 (scientist), 世界(world),首例(first case), 深圳(Shenzhen), 国际(international), 伦理(ethics),基因组编辑(genome editing), 艾滋病(AIDS), 项目(project), 出生(birth), 医院(hospital), 争议(controversy),试验(experiment),演讲(speech), 研究 (research), 实验(experiment), 回应 (response),娜娜 (Nana) | 【基因峰会发布声明：贺建奎项目存缺陷 不应临床试验】在香港举办受到全球瞩目的第二届人类基因组编辑国际峰会迎来峰会的最后一天。峰会组委会主席、诺贝尔奖学者戴维-巴尔的摩代表组委会发布了“二届人类基因组编辑国际峰会组委会声明”。声明称，这一项目的缺陷包括医疗指示不足、研究方案设计不当、 ​ |
|  |  | 【首例免疫爱滋基因编辑宝宝诞生　「报告疑作假」医院急报案】基因编辑婴儿露露和娜娜11月在大陆诞生，引发各界哗然，除了是否符合伦理备受争议外，科学家如何取得研究许可及报告真实性引发外界质疑。深圳和美妇儿科医院总经理程珍27日表示，医院怀疑南方科技大学副教授贺建奎申请报告作假，目前已向警 ​ |
|  |  | 【深圳科创委回应资助#首例免疫艾滋病基因编辑婴儿#项目”：不属实】据中国临床试验注册中心消息，基因编辑婴儿项目经费或物资来源为深圳市科技创新自由探索项目。26日，深圳科技创新委员回音称，从未立项资助“CCR5基因编辑”、“HIV免疫基因CCR5胚胎基因编辑安全性和有效性评估”等自由探索项目。 ​ |

# **S1 Fig 1. Preprocessing steps for Weibo posts.**


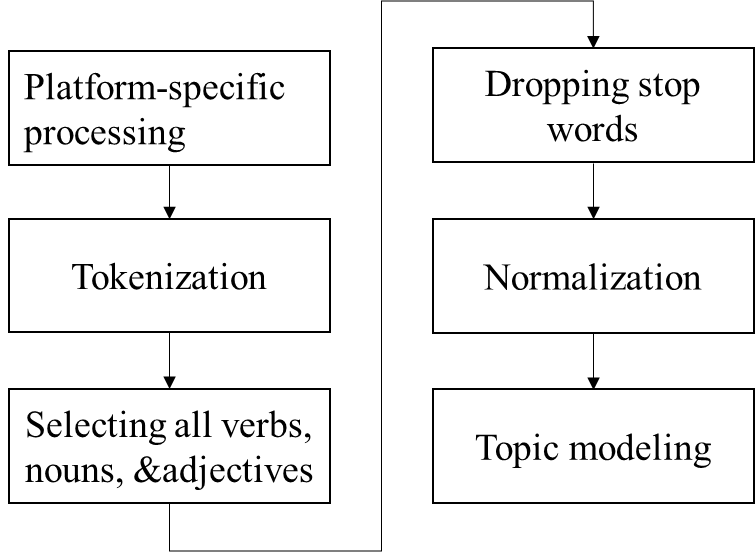


# **S1 Fig 2. Preprocessing steps for tweets.**


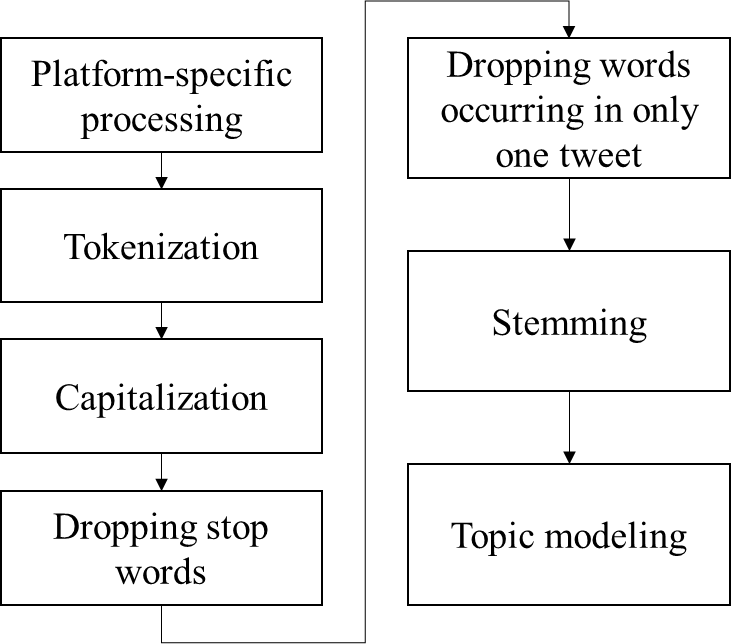


# **S1 Fig 3.** **The distributions for document lengths of tweets and Weibo posts.**


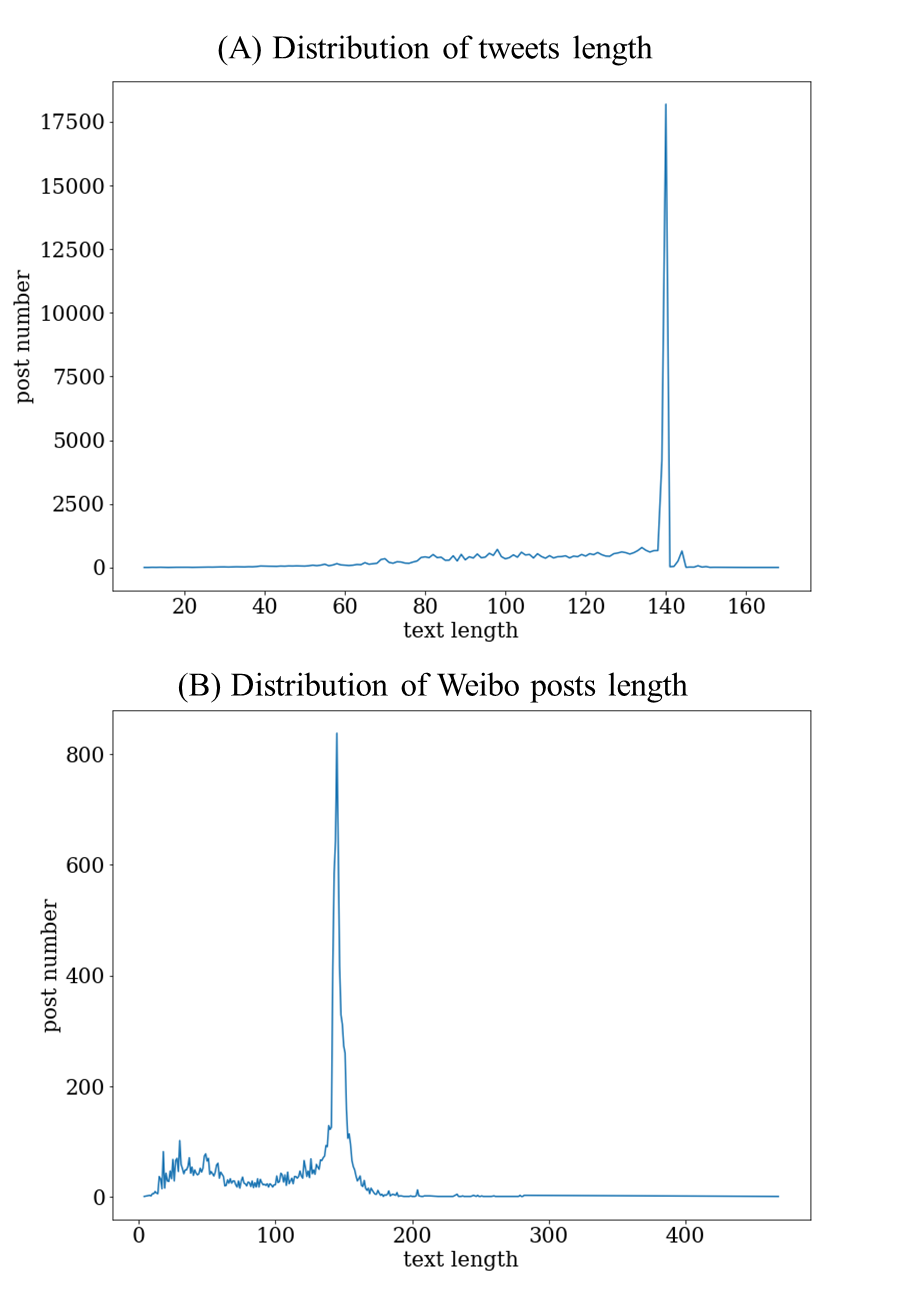


# **S1 Fig 4.** **Intertopic distance mapping for Weibo posts (left) and tweets (right).**


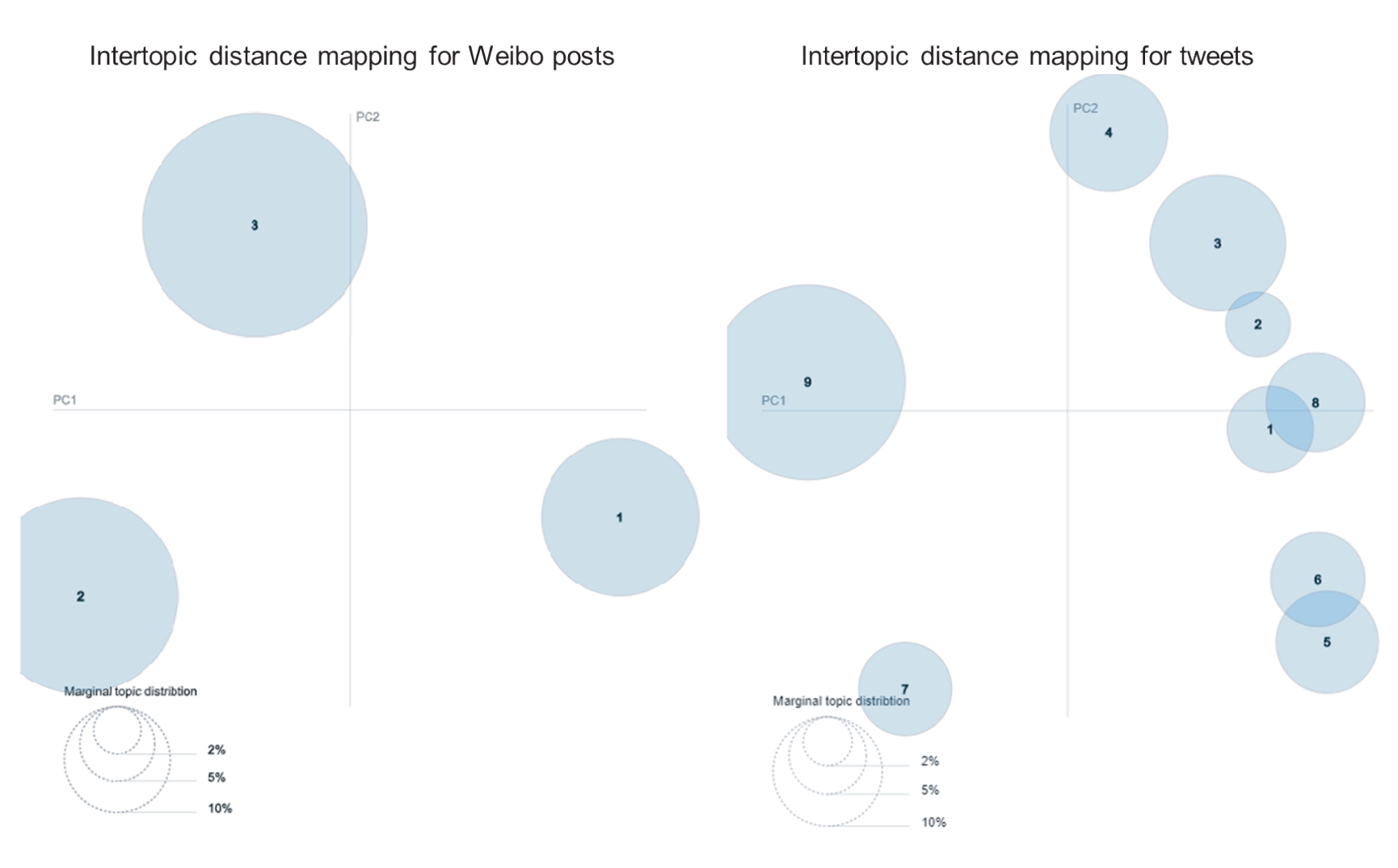


*Note.* The circle size represents the proportion of each topic within the corpus.

# **S1 Fig 5.** **Topic difference between verified accounts and unverified accounts.**


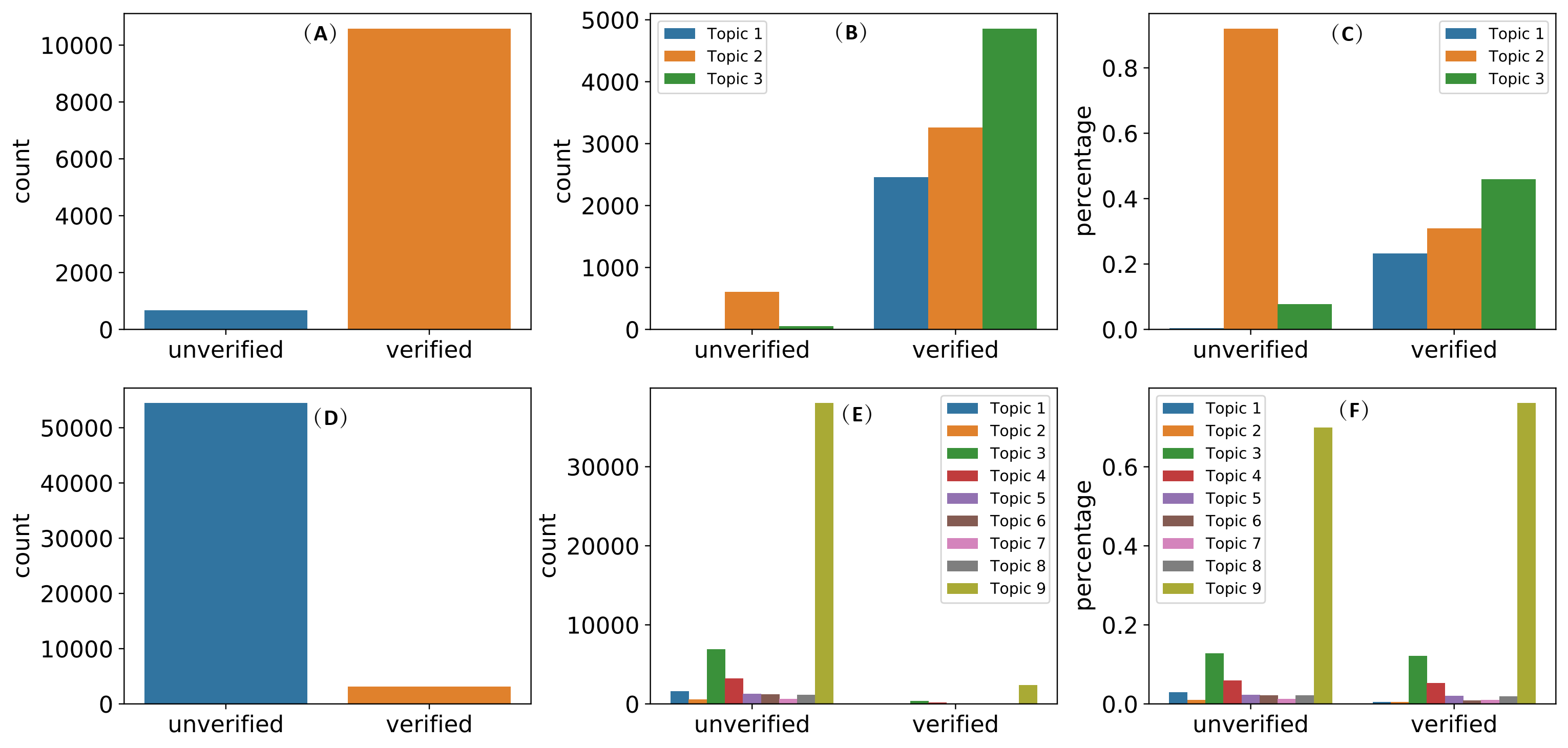


(A) user distribution on Weibo, (B) the number of three topics on Weibo, (C) the ratio of three topics on Weibo, (D) user distribution on Twitter, (E) the number of eight topics on Twitter, and (E) the ratio of eight topics on Twitter.
